# The study of Chinese EFL teachers' emotions in the digital transformation of education

**DOI:** 10.3389/fpsyg.2026.1855571

**Published:** 2026-10-14

**Authors:** Feng Wu, Youjie Hua

**Affiliations:** 1 Faculty of Education, Guangxi Normal University, Guilin, China; 2 School of Foreign Languages, Hezhou University, Hezhou, China

**Keywords:** Chinese EFL teachers, digital transformation of education, educational reform, teacher emotions, teacher goals

## Abstract

**Introduction:**

The rapid and continuing development of digital technologies has significantly reshaped the educational landscape, generating diverse emotional experiences among teachers. This study examined the emotional experiences of Chinese EFL teachers and the major antecedents of those emotions.

**Methods:**

This study employed an explanatory sequential mixed-methods design. First, questionnaire data collected from 296 university EFL teachers identified five primary emotions (love, joy, anger, sadness, and fear). Secondly, semi-structured interviews were conducted with 16 participants to explore the antecedents underlying these emotional experiences. The qualitative findings were subsequently integrated with the quantitative results and presented through a joint display.

**Results:**

The findings showed that EFL teachers experienced positive emotions more frequently than negative emotions. Of the five primary emotions, joy and love had the highest mean scores, followed by fear, anger, and sadness, respectively. The findings further showed that teachers' emotions were closely associated with their professional goals and students' performance. Several antecedents were found to shape these emotional experiences. The flexibility, interactivity, and innovative opportunities introduced by digital transformation enhanced EFL teachers' sense of love for their work. Likewise, the efficiency and abundance of resources provided by digital tools and platforms contributed to their sense of joy. In contrast, students' misconduct and dishonest behaviors often provoked feelings of anger. Teachers experienced sadness when confronted with students' lack of critical thinking and when they felt inadequately supported in their work. Fear was primarily driven by peer pressure, time constraints, and the threat of being replaced by artificial intelligence.

**Discussion:**

The study discussed the implications of these findings and proposed recommendations for emotion regulation and emotional support for EFL teachers in China and comparable educational contexts.

## 1 Introduction

The rapid advancement of digital technology is significantly reshaping the global educational landscape. This transformation represents a major shift in educational practice, characterized by the integration of smart devices, artificial intelligence (AI), virtual and augmented reality (VR/AR), and data-driven platforms into teaching and learning environments (OECD, 2021; Timotheou et al., 2023). For teachers, this transformation presents both unprecedented opportunities and complex challenges. On the one hand, digital technologies offer new possibilities for pedagogical innovation and improved instructional efficiency (Samuelsson, 2025; Thumvichit, 2025). On the other hand, they require teachers to rapidly adapt to emerging technical skills, evolving classroom dynamics, and changing student expectations (Heilporn and Lakhal, 2021; Kamalov et al., 2023). Central to this process of adaptation are teachers' emotional experiences, an important yet relatively underexplored aspect of educational digitization. Emotions are fundamental to the teaching profession (Hargreaves, 1998a; Zembylas, 2003), as they influence teachers' motivation, resilience, and professional identity (Scott and Sutton, 2009).

Compared with teachers in many other disciplines, English as a foreign language (EFL) teachers may encounter discipline-specific challenges and emotional experiences in the digital era (Gu et al., 2022; Liu and Chu, 2022). On one hand, digital tools help enhance language teaching and increase productivity in language educational settings (Ekizer, 2025; Liu and Chang, 2024). Evolving instructional modalities has the potential to evoke teacher emotions. Gu et al. (2022) found that EFL teachers experienced positive emotions such as satisfaction, love, and happiness in this process, as they could clearly monitor students' learning progress via online platforms, which also brought great convenience to formative assessment. On the other hand, a range of conventional language-instruction functions, such as training in translation, proofreading, and pronunciation correction, are increasingly being displaced by artificial intelligence and digital technologies (Pack and Maloney, 2023). As a result, some EFL teachers face growing uncertainty regarding their future professional roles. Concerns about whether language teachers might eventually be replaced by intelligent technologies have generated pressure, anxiety, and other complex emotional responses. Consequently, EFL teachers are experiencing a mixture of positive and negative emotions as they navigate the ongoing digital transformation of education (Shen and Guo, 2024).

Given that teacher emotions are culturally specific (Mesquita and Albert, 2007), Chinese EFL teachers' emotions amid digital educational transformation could exemplify the complexity of emotions experienced by language teachers in educational reforms. An investigation into the Chinese context advances our understanding of teacher emotion across diverse sociocultural settings and broadens scholarship concerning how teacher emotions support sustainable professional development. Adopting a mixed-methods design, this study explores the emotional experiences of 296 university EFL teachers engaged in digital teaching. More importantly, this study explores EFL teachers' overall emotional profiles across five primary emotions and the major antecedents that trigger each emotion. This study can contribute new insights into the ongoing inquiry into teacher emotions in educational reform and provide practical implications for university EFL teachers and administrators to foster teachers' emotional development.

## 2 Literature review

### 2.1 Study on teacher emotions

Over time, the concept of teacher emotions has evolved from being viewed solely through psychological theories to being understood through multiple perspectives, including social constructivism and poststructuralism (Zembylas, 2005). From a psychological standpoint, teacher emotions were predominantly regarded as subjective feelings and private psychological states (Sutton and Wheatley, 2003). However, this perspective does not fully capture the interaction between teachers' internal experiences and the external environment in which they work.

Recognizing this limitation, Schutz et al., 2006, p. 344 define emotions as “socially constructed, personally enacted ways of being that emerge from conscious and/or unconscious judgments regarding perceived successes at attaining goals or maintaining standards or beliefs during transactions as part of social-historical contexts.” This definition emphasizes that emotions arise through the interaction between individuals and their environments. In other words, teachers' emotional experiences are shaped by a combination of internal factors, including personal goals and beliefs, and external influences, such as institutional expectations, power relations, and broader sociocultural factors. Teachers often use their goals as reference points for evaluating how closely their current experiences align with their professional ideals, beliefs, and identities (Schutz and DeCuir, 2002).

Drawing on this perspective, the present study examines EFL teachers' emotions by considering not only the psychological factors that influence them but also the socio-cultural contexts in which they are embedded. It further explores teachers' reflective experiences, and hoe they interpret the meaning of their roles in the midst of the ongoing digital transformation of education.

### 2.2 Study on classification of emotions

Emotions have been categorized in many ways based on different criteria. One common approach is to distinguish between basic and compound emotions according to their level of complexity. Basic emotions are considered innate human feelings that serve essential life functions and form the foundation for more complex emotional experiences. Scholars have commonly identified joy, fear, anger, surprise, and sadness as primary or basic human emotions (Ekman, 1984, 1992). Conversely, compound emotions emerge through the combination, blending, or transformation of primary emotions (Kemper, 1987). They are generally more complex, shaped by cognitive processes, and influenced by contextual factors. Examples of compound emotions include guilt, shame, jealousy, pride, anxiety, and disappointment. For instance, guilt may arise from a combination of sadness, fear, and self-reproach.

Another widely used approach classifies emotions according to their valence, distinguishing between positive and negative emotions (Watson and Tellegen, 1985). Positive emotions typically include joy, satisfaction, pride and excitement, while negative emotions include anger, frustration, anxiety and sadness (Hargreaves, 1998b; Sutton and Wheatley, 2003). Although some scholars have criticized this classification for oversimplifying the complex nature of emotions (Sutton and Wheatley, 2003), it has nonetheless provided an important foundation for subsequent research on emotional categorization (Chen, 2016).

Building on these earlier approaches and emotional classifications, Parrott (2001) proposed a hierarchical tree structure that organizes emotions into primary, secondary, and tertiary levels. At the foundational level, the framework identifies six basic emotions: love, joy, surprise, anger, sadness, and fear. These primary emotions provide the basis for more precise secondary emotions. For example, love encompasses emotions such as affection, lust, longing, and cheerfulness, while secondary emotions can be further divided into even more sophisticated tertiary emotions. This hierarchical framework provides a detailed and comprehensive taxonomy of emotional experiences, ranging from broad primary emotions to highly specific emotional states, making it a valuable tool for emotion research.

Based on the above emotion categories, the present study adopts positive and negative emotion classifications to examine the emotional valence of EFL teachers. Additionally, a tree-structure of emotions comprising five primary emotions—love, joy, anger, sadness, and fear—is used to frame their emotions amid the digital transformation of education^1^.

### 2.3 Study on teacher emotions in educational reform

Teachers often experience strong emotional reactions during periods of educational reform, as such reforms typically bring significant changes to teaching practices and professional expectations (Fullan, 2005). Teacher emotions and educational reform are closely interconnected. On one hand, educational reform directly influences teachers' emotional experiences. Some researchers argue that due to the ambiguity and uncertainty inherent in educational reform, coupled with differences in teachers' personal experiences and capabilities, teachers may experience anxiety (Scott and Sutton, 2009) and pressure (Gao and Cui, 2022; MacIntyre et al., 2019) when confronting complex transformative scenarios. Similarly, Kelchtermans (2005) noted that educational reform often requires teachers to adjust their mindsets, modify their working practices, and adjust to new patterns of professional competition, making them feel vulnerable. For example, Lee et al. (2014) found that when teachers were required to redefine their professional roles and identities during educational reform, they experienced a range of emotions, including positive feelings like satisfaction and joy, as well as negative feelings such as pain, helplessness, and anxiety. On the other hand, teachers' emotions can significantly influence the success of educational reform. These emotions influence reform by affecting teachers' sustainable professional development. They deepen or impede teachers' understanding of reform, shape their professional identities, and motivate them to take action (Gu et al., 2022).

Compared with general educational reforms, integrating digital technology into education has the potential to thoroughly transform teaching and learning practices (Liu et al., 2023) and thereby exert profound impacts on teachers' emotions. A body of existing research has examined teachers' emotional struggles amid digital transformation. For instance, Feng et al. (2025) identified digital stress experienced by teachers as they adapt to new technologies and challenges brought by educational informatization and inclusive education. Dong et al. (2025) found that university teachers suffer from technological anxiety rooted in fears of job replacement and doubts over teaching quality. Since the advent of AI has sparked growing scholarly interest in exploring its effectiveness in language education (Gao and Cui, 2022), EFL teachers' emotional responses in digital contexts deserve more targeted research attention. Liu and Chang (2024) examined EFL teachers' emotional experiences in the AI teaching era and confirmed that positive emotions play a central role in fostering teachers' adaptive expertise. In online teaching scenarios, Gu et al. (2022) investigated five EFL teachers' emotional experiences during livestream instruction and revealed that teachers' emotions emerge from the interaction between personal teaching goals and external teaching environments. These emotional states further shape teachers' professional identities and motivate their instructional behaviors. To address emotional challenges, Gu et al. (2026) adopted Gross (1998) process model of emotion regulation as the analytical framework. Their findings revealed a wide spectrum of context-specific emotion regulation tactics deployed by teachers, including problem-solving coping, peer help-seeking, interpersonal empathy, expressive suppression, and enduring tolerance.

While existing research has offered meaningful observations regarding EFL teachers' emotional struggles within digital education contexts, limited work has simultaneously explored their full range of EFL teachers' emotions (both positive and negative) during educational digital transformation, and that little is known about the relative levels at which these emotions are endorsed and the antecedents that give rise to them. Drawing upon previous literature, the current work examines EFL teachers' overall emotions and their underlying reasons amid educational digital transition. The study addressed the following research questions (RQs):

RQ1. What are the overall levels of Chinese EFL teachers' primary emotions (joy, love, anger, fear, and sadness) amid digital transformation?

RQ2. What are the major antecedents of each primary emotion?

## 3 Method

Drawing from a series of studies on teacher emotions, this study employed an explanatory sequential mixed methods design (Creswell and Plano Clark, 2018). Quantitative data were first collected via a questionnaire to identify the levels and patterns of EFL teachers' emotions. Qualitative semi-structured interviews were conducted to systematically explain the antecedents underlying the quantitative findings.

### 3.1 Participants

This study was conducted from June to November in 2025. Convenience sampling, which can save time, money and effort in reaching participants, was used in this study. A total of 296 EFL teachers from various universities participated in the study, including 102 males (34.46%) and 194 females (65.54%). Regarding educational qualifications, 62 held a Ph.D. (20.95%), while 234 held a master's degree or below (79.05%). In terms of age, 43 participants were under 30 (14.53%), 119 were aged 31–40 (40.20%), 98 were aged 41–50 (33.11%), and 36 were over 51 (12.16%). With respect to academic ranks, 161 were lecturers (54.39%), 109 were associate professors (36.82%), and 26 were professors (8.78%) (see Table 1). All participants were fully informed about the study's purpose and procedures, as well as their rights regarding voluntary participation, withdrawal, and confidentiality. Written informed consent was obtained from all participants involved in the study.

**Table 1:** Demographic status of the participants.

Variable	Categories	Number	Percentage (%)
Gender	Male	102	34.46
	Female	194	65.54
Educational qualification	Doctoral degree	62	20.95
	Master's degree or below	234	79.05
Age	Under 30	43	14.53
	Ages 31–40	119	40.20
	Ages 41–50	98	33.11
	Age 51 and older	36	12.16
Academic rank	Lecturer	161	54.39
	Associate professor	109	36.82
	Professor	26	8.78
Total		296	100.00

### 3.2 Instrument

#### 3.2.1 Questionnaire

The questionnaire consisted of two sections. The first section collected participants' demographic information, including gender, age, years of teaching, academic rank, and frequency of using digital teaching tools and platforms. The second section aimed to understand EFL teachers' five primary emotions (love, joy, sadness, anger, and fear) regarding educational digital transformation, using a five-point Likert scale ranging from 1 (Strongly Disagree) to 5 (Strongly Agree).

The EFL Teacher Emotion Scale used in the study was adapted from Chen (2016) Teacher Emotion Inventory (TEI), which was developed based on Parrott (2001) tree structure of emotions as the framework for designing the questions. To ensure its relevance to the context of educational digital transformation, 16 EFL university teachers were interviewed to explore their emotional experiences during this transformation. Building on the original TEI scale and interview data, we designed an initial scale including 50 items for this study. After that, four university specialists holding doctoral degrees or professorial titles were called to evaluate the instrument. The specialists rated each item on two dimensions: relevance (1 = not relevant, 2 = somewhat relevant, 3 = highly relevant) and necessity (1 = not necessary, 2 = somewhat necessary, 3 = essential). The item-level Content Validity Index (I-CVI) was calculated as the proportion of experts rating an item as highly relevant, and the Content Validity Ratio (CVR) was recorded to assess item necessity. Items with I-CVI ≥0.78 and CVR ≥0.75 were retained. The scale-level CVI (S-CVI/Ave) was 0.86 for the initial 50-item pool. Inter-rater agreement was examined using Fleiss' Kappa, yielding values of 0.56 for relevance ratings and 0.74 for necessity ratings, indicating moderate to substantial agreement among the experts. Based on the content validity indices and expert feedback, three items were deleted and 22 existing items were revised.

Following the experts' review, a revised 47-item TEI was retained for further analysis. An exploratory factor analysis (EFA) was conducted on the 47-item pool using principal axis factoring with Promax rotation, with the number of factors fixed at five. The Kaiser-Meyer-Olkin (KMO) measure of sampling adequacy was 0.886, which exceeded the recommended threshold of 0.70. Bartlett's test of sphericity was also significant (χ^2^ = 6325.345, df = 1081, *p* < 0.001), confirming that the data were appropriate for factor analysis. Fourteen items were subsequently removed because they had factor loadings below 0.40 or showed cross-loadings. A second EFA conducted on the remaining 33 items revealed a five-factor structure that accounted for 54.90% of the total variance. Compared with Chen (2016) original 26-item TEI scale, the adapted scale comprised 33 items, with three additional items in Love, two in Joy, one in Sadness, and one in Anger, while the Fear dimension retained the same seven items. These additional items were included based on two sources of evidence. First, the interview data revealed that EFL teachers experienced a wide range of emotions in digital teaching contexts, particularly regarding joy and love, which were not fully captured by the original scale. Second, the subsequent exploratory factor analysis confirmed the psychometric adequacy of these newly added items, supporting their retention in the adapted scale.

The Cronbach's alpha coefficients for the five emotional dimensions of love, joy, sadness, anger, and fear were 0.83, 0.82, 0.92, 0.85, and 0.91, respectively, indicating good to excellent internal consistency across the five dimensions.

Consistent with the positive–negative classification of emotions proposed by Watson and Tellegen (1985), love and joy were grouped into positive emotions, while sadness, anger, and fear were grouped into negative emotions. The final instrument, therefore, included 16 items measuring positive emotions (7 for love and 9 for joy) and 17 items measuring negative emotions (5 for sadness, 5 for anger, and 7 for fear).

#### 3.2.2 Semi-structured interviews

Semi-structured interviews were conducted to complement the questionnaire findings and provide further insight into the issues identified. The interview questions were adapted from Chen (2020) and revised to reflect the context of educational digital transformation. The initial version of the questions was piloted with two university EFL teachers. Based on their feedback, minor revisions were made to improve clarity and wording before the final version was developed. The interview questions were as follows: (1) Please describe the emotions you have experienced during the digital transformation of education, in terms of love, joy, anger, sadness, and fear. (2) Please describe those emotional situations. (3) Please share five examples of emotional situations that impressed you the most in the context of educational digital transformation.

Sixteen participants were selected for in-depth interviews to provide rich, contextual insights into their emotions in the digital transformation of education. The interview data was used both in the previous design of questionnaire revision and formal qualitative analysis, while the two pilot interview data were not incorporated into the official interview dataset. The interviewees represented a wide range of genders, educational levels, ages, academic ranks and the frequency of using teaching digital tools and platforms. Specifically, the 16 participants consisted of 7 males and 9 females aged 28-54, with teaching experience ranging from 1 to 29 years. Among them, 12 held master's degrees and 4 held doctoral degrees. Their academic ranks covered teaching assistant, lecturer, associate professor, and professor. In terms of the frequency of using teaching digital tools and platforms, eight participants were high-frequency users, six used digital tools and platforms several times a week, and only one participant reported occasional use. Each session lasted 30 to 60 min. With the participants' consent, all interviews were audio-recorded and later transcribed for analysis. The demographic profiles of the participants are presented in Table 2.

**Table 2:** Demographic information of interviewees.

Participant	Gender	Age	Years of teaching	Education background	Academic rank	Frequency of using teaching digital tools and platforms
P1	Male	32	6	Master's degree	Lecturer	High-frequency
P2	Female	33	6	Master's degree	Lecturer	Several times a week
P3	Male	33	5	Master's degree	Lecturer	high-frequency
P4	Female	29	4	Master's degree	Lecturer	Several times a week
P5	Female	32	5	Master's degree	Lecturer	Several times a week
P6	Female	29	2	Master's degree	Lecturer	High-frequency
P7	Male	40	12	Doctoral degree	Associate Professor	High-frequency
P8	Female	43	20	Master's degree	Lecturer	High-frequency
P9	Male	46	26	Master's degree	Associate Professor	High-frequency
P10	Female	54	29	Master's degree	Associate Professor	Several times a week
P11	Male	43	20	Doctoral degree	Professor	High-frequency
P12	Female	35	7	Master's degree	Lecturer	Several times a week
P13	Male	49	21	Master's degree	Lecturer	High-frequency
P14	Female	32	10	Doctoral degree	Lecturer	High-frequency
P15	Male	38	2	Doctoral degree	Lecturer	Occasional use
P16	Female	28	1	Master's degree	Teaching Assistant	Several times a week

### 3.3 Data collection and analysis

Ethical approval for the study was obtained from the first author' s affiliated university. The questionnaire links were distributed to full-time university EFL teachers across multiple higher education institutions across China using purposive and snowball sampling. Teachers within the authors' academic network were initially contacted via email and WeChat. These initial participants were then encouraged to share the survey with other eligible university English instructors. All respondents were fully informed of the study's purpose, the confidentiality of their answers, and their right to voluntary participation. After data cleaning, 296 valid responses were retained for analysis. Of these, 129 responses (43.58%) were collected from Guangxi, a southwestern Chinese province where the authors are based. SPSS 27.0 was used to analyze the questionnaire data.

For the qualitative phase, inductive thematic analysis (Braun and Clarke, 2006) was adopted, and NVivo 15 was used to code and analyze the interview data. Parrott (2001) tree structure of emotions was used to classify the emotional categories expressed by EFL teachers in the interview transcripts. For example, if a participant mentioned, “I am worried that artificial intelligence may replace part of my instructional duties,” worry counts as a tertiary emotion falling under the broader category of fear, so this emotional response was coded under fear. Following the six-phase thematic analysis procedure proposed by Braun and Clarke (2006), the researchers first familiarized themselves with all transcripts, generated open codes sentence by sentence, then grouped codes to search for candidate themes, reviewed and refined themes, defined and named themes, and finally constructed the narrative supported by participant quotations. Open coding was implemented sentence by sentence, followed by selective coding to extract core themes. For any coding discrepancies, the two authors held repeated discussions to reach consensus; unresolved disputes were submitted to two external professionals to establish coding validity. Cohen's Kappa was computed to determine the level of agreement between coders. The analysis produced a Kappa value of 0.83, indicating a strong level of inter-coder reliability (Cohen, 1960).

## 4 Findings

### 4.1 Findings on overall levels of EFL teachers' emotions

The descriptive statistics presented in Table 3 provide an overview of the emotional profiles of Chinese EFL teachers during educational digital transformation. Overall, the teachers reported moderately high levels of emotional experiences, as the mean scores for all sub-dimensions were above the neutral midpoint of 3 on the 5-point Likert scale. When the emotions were considered in terms of positive and negative valence, positive emotions recorded yielded much higher mean score (*M* = 4.04, *SD* = 0.61) than negative emotions (*M* = 3.67, SD = 0.68). This suggests that positive emotions were somewhat more prevalent among the EFL teachers

**Table 3:** Results of overall levels of EFL teacher emotions.

Sub-dimensions	No. of Items	Mean	Standard Deviation	95% Confidence Interval	
				Lower Limit	Upper Limit
Positive emotions	16	4.04	0.61	3.97	4.11
Love	7	4.01	0.65	3.93	4.08
Joy	9	4.07	0.64	3.99	4.15
Negative emotions	17	3.67	0.68	3.60	3.75
Anger	5	3.64	0.78	3.55	3.73
Sadness	5	3.57	0.81	3.48	3.66
Fear	7	3.77	0.77	3.68	3.85

The mean score for each sub-dimension was computed as the average of all items belonging to that dimension.

Among the five primary emotions, joy (*M* = 4.07, *SD* = 0.64) and love (*M* = *4*.01, *SD* = 0.65) emerged as the top two, ranking first and second, respectively. They were followed by fear (*M* = 3.77, *SD* = 0.77), anger (*M* = *3*.64, *SD* = 0.78), and sadness (*M* = 3.57, *SD* = 0.81) in descending order. Notably, joy stood out as the most salient positive emotion, while fear was the most prominent negative one.

### 4.2 Findings on major antecedents of EFL teachers' emotions

Among the five primary emotions, the sub-dimensions with the highest mean scores highlighted the emotions that EFL teachers experienced most frequently. The semi-structured interviews provided deeper insights into these findings by explaining the reasons behind the reported emotions. In addressing RQ2, the integration of quantitative and qualitative revealed the key factors shaping each of the five primary emotions, as presented below.

#### 4.2.1 Flexibility, interactivity, and innovation fostering EFL teachers' love

As shown in the joint display (Table 4), across all items in the love sub-dimension, the means of Love 1, Love 3, and Love 7 (*M* = 4.09, 4.10, and 4.22, respectively) were higher than the overall average (*M* = 4.03), indicating that these items played a greater role in shaping this emotion. Thematic analysis identified three interrelated antecedents underlying the quantitative findings.

**Table 4:** Joint display integrating quantitative and qualitative findings for main antecedents of each primary emotion.

Emotion Dimension (*M, SD*)	Quantitative Salient Items (*M, SD*)	Qualitative themes	Representative Quote (ID)
Love (4.01, 0.65)	Love 1 (4.09, 0.78)	**Theme 1: Temporal and spatial flexibility**. Digital tools remove time/space barriers, enabling pre-class tasks that free up class time for deeper interactions.	“I use the SuperStar platform to assign tasks to students in advance, which saves a significant amount of class time for in-depth interactions. This has inspired my passion for teaching.” (P16)
	Love 3 (4.10, 0.82)	**Theme 2: Enhanced classroom interactivity**. Digital platforms transform one-way lectures into multi-directional exchanges, creating reciprocal emotional dynamics.	“The students have been very active and enthusiastic in the online course—I often see their likes and comments, which has sparked my passion for teaching.” (P1)
	Love 7 (4.22, 0.77)	**Theme 3: Innovative instructional design**. AI tools and platform features enable teachers to transform static lesson plans into dynamic learning experiences.	“I usually use AI to create teaching slides... AI can also generate images and videos suitable for teaching, which greatly enhances the interest and innovation of my classes.” (P12)
Joy (4.07, 0.64)	Joy 1, Joy 5 (4.21, 0.80; 4.23, 0.76)	**Theme 1: Digital efficiency and workload reduction**. Automation of routine tasks (grading, planning, slides) reduces workload, bringing relaxation and satisfaction.	“With the advent of AI tools, the task [revising syllabi] became easy, and I felt quite happy about using digital tools.” (P8)
	Joy 2 (4.18, 0.81)	**Theme 2: Resource accessibility and enriched content**. AI platforms and open educational resources provide quick access to high-quality materials, enriching instruction and bringing joy.	“By embedding national first-class online course resources, I greatly enriched the depth and breadth of the course content... a result that made me feel genuinely happy.” (P8)
Anger (3.64, 0.78)	Anger 1 (3.70, 0.98)	**Theme 1: Students' misuse of digital tools for entertainment**. Smartphones intended for learning are used for entertainment, causing distraction and frustration.	“I put so much effort into preparing my lessons, but some students aren't listening at all—they just keep playing with their smartphones. I really feel angry and frustrated.” (P10)
	Anger 4 (3.86, 1.01)	**Theme 2: Academic dishonesty and AI plagiarism** AI-enabled plagiarism undermines academic integrity and erodes teachers' trust.	“The paper showed clear signs of AI generation... When I asked him to explain the key parts, he couldn't answer. I was angry about his dishonesty and deception.” (P7)
Sadness (3.57, 0.81)	Sadness 1 (3.71, 0.99)	**Theme 1: Students' reduced independent thinking** AI encourages reliance on ready-made answers rather than critical thinking, disappointing teachers.	“Students immediately take out their phones to search for answers without even thinking. This hinders students' moral cultivation and ability development.” (P3)
	Sadness 5 (3.66, 0.97)	**Theme 2: Insufficient support for teacher digital literacy** Despite government emphasis, teachers lack training and resource support, feeling helpless.	“I did not know how to better integrate digital technology into my teaching. Besides, I lacked sufficient guidance and support, which made me feel frustrated.” (P8)
Fear (3.77, 0.77)	Fear 1 (3.78, 1.09)	**Theme 1: Peer pressure in digital literacy development** Teachers feel pressure when observing colleagues' proficiency while struggling to keep up.	“I really admired the teacher's ability to use digital tools effectively, but I felt pressured because those technologies seemed difficult for me to master.” (P11)
	Fear 3 (3.97, 1.02)	**Theme 2: Time scarcity** Constant platform updates consume time and energy, creating anxiety about balancing development with teaching.	“These platforms had varying requirements, and their functions were constantly being updated. I had pressure to constantly explore and learn, which consumed a great deal of time.” (P5)
	Fear 6 (3.87, 1.04)	**Theme 3: AI threat and job stability concerns** AI replacement and declining language major enrollment threaten professional identity and career stability.	“Due to AI translation tools, the roles held by foreign language talents are being displaced. I am concerned that EFL teachers with lower qualifications could lose their jobs.” (P12)

This joint display follows an explanatory sequential (QUAN → QUAL) logic. Quantitative results identify salient items under each primary emotion; qualitative interview data explore their contributing antecedents. M, mean; SD, standard deviation. Values in parentheses under Salient Items are mean scores and standard deviations, respectively.

Firstly, EFL teachers' love for teaching was strengthened by the ability of digital tools to transcend the limitations of time and space, as illustrated in Love 1. Three interviewees (18.75%) emphasized that digital platforms removed temporal and spatial barriers, enabling pre-class task assignment that freed up class time for deeper interactions. As one participant explained, using the SuperStar platform to assign tasks in advance “saves a significant amount of class time for in-depth interactions” and “has inspired my passion for teaching” (P16). This theme suggests that the flexibility provided by digital tools can ease teachers' time pressure and create space for more meaningful engagement with teaching, thereby strengthening their emotional connection to their work.

Secondly, EFL teachers' love for teaching was associated with the interactivity enabled by digital platforms, as shown in Love 3. Digital teaching platforms can transform traditionally one-way lectures into more interactive, multi-directional exchanges through features such as real-time polling, which allows students to share their opinions instantly and increases classroom interaction. This was further demonstrated in the interviews, where two participants (12.5%) attributed their renewed enthusiasm for teaching to these interactive learning environments. One teacher noted that students who were “shy to ask questions during class” became active online, and their likes and comments “sparked my passion for teaching this course” (P1). This finding indicates that digital interactivity creates a reciprocal emotional dynamic—student engagement reinforces teacher enthusiasm, which in turn further promotes student participation.

Thirdly, EFL teachers' love for teaching was linked to the opportunities for innovation provided by digital tools and resources. These technologies enable teachers to move beyond traditional and relatively static lesson plans and develop more engaging and dynamic learning experiences. One participant reported that AI “greatly enhances the interest and innovation of my classes” and “has strengthened my love for teaching” (P12). This theme illustrates how digital innovation can enable teachers to rethink their pedagogical practices, contributing to a stronger sense of professional fulfillment and emotional investment in teaching.

#### 4.2.2 Digital efficiency and resource accessibility cultivating EFL teachers' joy

As shown in the joint display (Table 4), the mean scores for Joy 1, Joy 2, and Joy 5 (*M* = 4.21, 4.18, and 4.23, respectively) were higher than the overall mean for the joy dimension (*M* = 4.07). This indicates that these three aspects made relatively important contributions to EFL teachers' reported joy. The thematic analysis of the qualitative data provided further insight into these quantitative findings.

Firstly, EFL teachers' sense of joy was associated with the greater efficiency enabled by digital technologies, particularly in relation to Joy 1 and Joy 5. Digital tools and platforms help automate routine tasks, including grading assignments, assigning homework, preparing lesson plans, and developing teaching slides. By reducing the time and effort required for these activities, such technologies help teachers manage their workload more efficiently and devote greater attention to meaningful teaching activities. Five interviewees (31.25%) specifically reported that being relieved of repetitive tasks generated feelings of happiness and satisfaction. One participant explained that AI tools transformed syllabus revision, which had previously been “time-consuming and laborious,” into an “easy” task, leaving him “quite happy about using digital tools” (P8). This finding suggests that digital efficiency not only alleviates teachers' workload but also creates greater emotional space for positive experiences in their professional work.

Secondly, EFL teachers' joy was enhanced by the wide range of resources made accessible through digital technologies, as reflected in Joy 2. AI platforms such as ChatGPT and DeepSeek, along with open educational platforms like Superstar and MOOCs, give teachers quick access to high-quality teaching materials, reducing the time spent searching for resources and supporting more effective instruction. One participant explained that embedding national first-class online course resources “greatly enriched the depth and breadth of the course content” and enhanced teaching quality, a result that made her “feel genuinely happy” (P8). This finding indicates that the accessibility of rich digital resources empowers teachers to improve their instructional quality, which in turn generates professional satisfaction and joy.

#### 4.2.3 Student misconduct and dishonesty provoking EFL teachers' anger

As shown in the joint display (Table 4), the mean scores for Anger 1 and Anger 4 (*M* = 3.70 and 3.86, respectively) were higher than the overall mean for the anger dimension (*M* = 3.64). This indicates that these two items were relatively important in shaping EFL teachers' experiences of anger. The qualitative findings provided further explanation of these quantitative results.

Firstly, EFL teachers' anger was associated with students' misuse of digital tools, as reflected in Anger 1. Although smartphones were introduced to facilitate learning and classroom interaction, some students used them primarily for entertainment, resulting in distraction and reduced engagement during lessons. Such behavior was a source of frustration for teachers. Four interviewees (25%) reported anger in such situations. One teacher expressed frustration that “some students aren't listening at all—they just keep playing with their smartphones” (P10), while another described a student who “persistently watched short videos and ignored my instructions” despite repeated reminders (P7). This finding suggests that the mismatch between the intended educational use of digital tools and their actual misuse by students creates a sense of wasted effort that triggers teachers' anger.

Secondly, EFL teachers' anger was related to students' academic dishonesty, particularly the misuse of AI tools for plagiarism and deceptive practices, as reflected in Anger 4. Such practices were perceived as undermining academic integrity and interfering with the teaching and learning process, thereby generating frustration and anger among teachers. Two interviewees (12.5%) raised this concern. One participant described discovering an AI-generated paper where the student “couldn't answer my questions,” which made her “angry about his dishonesty and deception” (P7). This finding indicates that AI-enabled academic dishonesty not only violates ethical standards but also erodes teachers' trust in students' work, intensifying feelings of anger.

#### 4.2.4 Student disengagement and insufficient TDL support inducing EFL teachers' sadness

As shown in the joint display (Table 4), across all items in the sub-dimension of sadness, the means of Sadness 1 and Sadness 5 (*M* = 3.71 and 3.76, respectively) were higher than the overall average (*M* = 3.57), indicating that these items played an important role in reflecting this emotion. Thematic analysis of the qualitative data complemented these quantitative findings.

Firstly, EFL teachers' sadness stemmed from students' reduced willingness to think and explore independently in digital learning environments, as shown in Sadness 1. The widespread use of AI tools has encouraged some students to rely on ready-made answers rather than engage in critical thinking, leaving teachers feeling disappointed about students' learning and development. Three interviewees (18.75%) expressed this concern. One teacher lamented that students “immediately take out their phones to search for answers without even thinking,” which “hinders students' moral cultivation and ability development” and undermines “the true value of education” (P3). This theme reflects a deeper concern: digital tools designed to enhance learning may paradoxically diminish students' cognitive engagement, leading teachers to feel saddened by the erosion of the broader purpose of education.

Secondly, EFL teachers' frustration and helplessness were associated with insufficient support for developing Teacher Digital Literacy (TDL), as shown in Sadness 5. In 2022, the Chinese Ministry of Education released *Teacher Digital Literacy*, which outlines requirements for teacher digital literacy across five dimensions: digital awareness, digital technology knowledge and skills, digital application, digital social responsibility, and professional development. Although EFL teachers recognized the importance of digital literacy and expressed a desire to meet these requirements, they reported insufficient access to relevant training resources and support. Two interviewees (12.5%) expressed this frustration. One participant explained that she “did not know how to better integrate digital technology” and “lacked sufficient guidance and support, which made me feel frustrated” (P8). This finding indicates that a gap between institutional expectations for digital literacy and the support available to teachers can create a sense of professional inadequacy that contributes to teachers' sadness.

#### 4.2.5 Peer pressure, time scarcity, and AI threat eliciting EFL teachers' fear

As shown in the joint display (Table 4), among all items in the fear sub-dimension, the mean scores for Fear 1, Fear 3, and Fear 6 (*M* = 3.78, 3.97, and 3.87, respectively) exceeded the overall mean (*M* = 3.77), indicating that these items were key indicators of teachers' fear. The qualitative findings provided further insight into the factors underlying these quantitative results.

Firstly, EFL teachers' fear stemmed from peer pressure, as reflected in Fear 1. Teachers experienced pressure when observing colleagues' proficient use of digital tools while they themselves struggled to keep pace. Two interviewees (12.5%) reported experiencing such peer pressure. One teacher admired a colleague's digital teaching demonstration but “felt pressured because those technologies seemed difficult for me to master” (P11). This theme suggests that digital transformation may create a competitive professional environment in which teachers compare their digital competence with that of their peers, generating anxiety about falling behind.

Secondly, EFL teachers' anxiety arose from insufficient time to develop their digital literacy, as indicated in Fear 3. The continuous introduction of new platforms and changing technological requirements consumed considerable time and energy, creating anxiety about balancing digital literacy development with routine teaching responsibilities. Two participants (12.5%) reported experiencing this form of time related anxiety. One participant explained that learning new platforms “consumed a great deal of time and energy” compared with traditional methods, which were “far less time-consuming and exhausting” (P5). Drawing on Rosa (2013) critical theory of social acceleration, this finding suggests that technological acceleration does not necessarily free teachers' time; rather, it may intensify their workload by requiring continuous adaptation to emerging technologies.

Thirdly, EFL teachers' anxiety stemmed from concerns about job stability as AI increasingly assumed some tasks traditionally performed by teachers, as reflected in Fear 6. With the growing integration of digital technologies into education, AI has begun to perform functions such as supporting language learning and providing feedback. This development led some teachers to question the future of the profession and their own career stability, generating uncertainty about their professional identity. Three interviewees (18.75%) expressed this concern. One participant noted that the university had stopped enrolling students in the translation major and that English-major quotas were declining, leading her to worry that “EFL teachers with lower academic qualifications and ranks like myself could potentially lose their jobs” (P12). This finding indicates that digital transformation not only reshapes teaching practices but may also heighten teachers' concerns about their professional futures, thereby generating anxiety about career stability.

In summary, the concise answer to RQ 2 was: (1) the flexibility, interactivity, and innovation brought by digital transformation enhanced EFL teachers' sense of professional love; (2) the efficiency and resources enabled by digital tools and platforms fostered EFL teachers' joy in teaching; (3) students' misconduct and deception triggered EFL teachers' feelings of anger; (4) students' laziness in independent thinking and teachers' lack of support in professional literacy development induced EFL teachers' sense of sadness; (5) peer pressure, time constraints, and the threat of replacement by AI elicited EFL teachers' fear. The joint display (Table 4) provides a comprehensive integration of these quantitative and qualitative findings.

## 5 Discussion and suggestions

### 5.1 Discussion

This study examined the overall emotional profiles of 296 university EFL teachers in China. The findings revealed that during the digital transformation of education, these teachers experienced more positive emotions (*M* = 4.04) than negative ones (*M* = 3.67). The results also suggest that teachers' emotions were closely tied to their professional goals. Drawing on Pekrun (2006) Control-Value Theory, this pattern can be understood through teachers' control and value appraisals: when digital technologies enhanced teaching efficiency, interaction, and productivity—enabling teachers to feel confident in achieving their instructional goals (high control) and perceiving these goals as professionally meaningful (high value)—they experienced higher levels of love and joy for teaching. In contrast, when technology created obstacles to these goals or threatened their professional identity, negative emotions became more prominent. This finding is consistent with previous research that teachers' emotions are closely related to teaching goals, which serve as standards for evaluating their success in professional practice (Schutz, 2014). Besides, beyond facilitating EFL teachers' goal achievement, digital tools also fostered a sense of agency in navigating pedagogical changes, which increased their positive emotions.

The findings also indicate that EFL teachers' emotions were closely associated with student performance, which was consistent with former studies (Chen, 2020; Cross and Hong, 2012; Han et al., 2023). When students used AI appropriately and improved their studies, EFL teachers felt positive emotions. Conversely, when students indulged in online entertainment, were reluctant to think independently, and resorted to plagiarism and deception, these behaviors triggered negative emotions in them.

Among the positive emotions, joy (*M* = 4.04, *SD* = *0*.61) emerged as the most strongly experienced emotion. This finding is consistent with earlier studies on Chinese teachers' primary emotions (Chen, 2016, 2020; Han et al., 2023), which likewise identified joy as the most prominent emotion. Previous research has attributed this to teachers' respected social status in China (Han et al., 2023) and to the greater sense of fulfillment that develops with increased teaching experience (Chen, 2020). In the digital age, EFL teachers report positive emotions because online platforms facilitate formative assessment (Gu et al., 2022). The present study extends these findings by showing that, greater teaching efficiency and easier access to abundant digital resources further enhanced teachers' sense of joy.

With regard to negative emotions, fear (*M* = 3.67, *SD* = *0*.68) emerged as the most frequently experienced negative emotion reported by EFL teachers. This differs from findings in traditional teaching contexts, where fear was typically the least prominent of the primary emotions (Chen, 2020; Han et al., 2023). One possible explanation is that digital transformation represents a comprehensive and complex educational reform, requiring teachers to continually update their educational philosophy, teaching methods, media, and assessment approaches (Akour and Alenezi, 2022). These demands inevitably create pressure and uncertainty. The prominence of fear among Chinese EFL teachers (which mainly included peer pressure and competence anxiety) as their strongest negative emotion is consistent with previous studies on teacher emotions during educational reform, in which teachers reported pressure and anxiety (Lee et al., 2014; Lee and Yin, 2011; Scott and Sutton, 2009).

Moreover, the digital age heightened people's concerns regarding job security, given that computerization and automation increased the risk of job displacement (Bond et al., 2018). Such risks spread across various occupations, with teachers-particularly language teachers—facing heightened exposure. English major graduates face an uncertain future as AI replaces basic skills (Zou, 2025). Consistent with these developments, many participants in the present study expressed concerns about the future of the EFL profession and the stability of their careers in the digital era. Besides, compared with conventional teaching contexts, EFL teachers experience distinct emotions such as anger and sadness. Their anger is triggered by students' misbehavior and dishonesty, while their sadness stems from students' reluctance to think independently and insufficient institutional support for teachers' professional literacy growth.

### 5.2 Suggestions

To enhance EFL teachers' emotional experience in the digital transformation of education, this paper offers recommendations centered on emotion regulation and emotional support to provide practical implications for university EFL teachers and administrators.

#### 5.2.1 EFL teachers' emotion regulation

Teachers with strong emotion regulation abilities are better able to manage their emotions and tend to experience higher levels of job satisfaction and wellbeing (Yin et al., 2016). Therefore, it's necessary for EFL teachers to employ emotion regulation strategies to proactively manage their positive emotions. Gross (1998) defined emotion regulation as “the processes by which individuals influence which emotions they have, when they have them, and how they experience and express them,” and distinguished between two broad approaches: antecedent-focused strategies, which occur before emotions arise, and response-focused strategies, which are used after emotions have been triggered. Building on this theoretical framework, the present study examines EFL teachers' emotion regulation practices and provides targeted suggestions for helping them manage the emotional challenges they encounter in their daily teaching practice.

For antecedent-focused regulation, EFL teachers may first need to reconsider how they perceive educational digital transformation. Rather than viewing technological change primarily as a threat, they can approach it as an opportunity for professional development and adapt positively to emerging pedagogical demands. At the same time, it is important to recognize that technology cannot replace the fundamental purpose of education. Education extends beyond the transmission of knowledge; it is grounded in human relationships, emotional connection, moral guidance, and meaningful interaction. Adopting this perspective can help reduce the pressure and anxiety associated with digital transformation.

For response-focused regulation, EFL teachers can seek emotional support when negative emotions arise (Gu et al., 2026). For instance, when facing technology adaptation stress or career development anxiety, they can confide in peers, family, or friends to find an emotional outlet. Communication with colleagues is particularly valuable because they understand the same professional challenges and can offer empathy, encouragement, and practical support. Such interactions help relieve emotional stress and strengthen teachers' confidence in their professional roles.

#### 5.2.2 Universities' emotional support

Teachers' emotions influence not only their wellbeing, teaching quality, and professional development but also the success of educational reform and school development. Teachers are more likely to take on risks in educational reform when they receive adequate emotional support (Kelchtermans, 2005). Therefore, universities should value teachers' emotions and professional identity and provide sufficient support for them during reform, especially in the comprehensive and systematic reform of digital transformation in education. Universities need to establish support systems in terms of technology, resources, and emotional care.

First, universities should strengthen digital infrastructure and improve support services for teachers. Inadequate hardware facilities or technical support may hinder teachers from implementing digital teaching, undermine the achievement of their goals, and trigger negative emotions among teachers. Many teachers expressed anger and helplessness when their needs for digital teaching were not met. To this end, universities must provide sufficient digital resources, including both hardware and software, and offer adequate professional personnel to help teachers resolve problems promptly. Resource and technological support are essential for facilitating teachers' goal achievement, thereby enhancing their positive emotions.

Second, universities should offer more systematic and flexible digital skills training. This study showed that many EFL teachers experience peer pressure and technological stress due to insufficient digital competencies. To address this issue, universities should provide EFL teachers with more flexible and diversified opportunities for digital training. Teachers could be given greater choice in selecting training programmes that are relevant to their teaching disciplines and aligned with their professional interests. As teachers develop the digital competencies required to meet institutional standards and instructional demands, their experiences of anger, sadness, and anxiety may gradually diminish. In addition, timely guidance and continuous support can help teachers address technological difficulties, alleviate negative emotional experiences, and build greater confidence in using digital.

## 6 Conclusion

This study explored the emotional experiences of EFL teachers using a mixed-methods approach, with particular attention to the antecedents shaping these emotions during the digital transformation of education. The findings contribute to a deeper understanding of EFL teachers' emotional experiences within the context of China's educational digital transformation, particularly by highlighting the relationship between teacher emotions, their professional goals, and students' performance. Moreover, these emotions had a lasting effect on teachers' professional development, particularly by strengthening their understanding of digital teaching.

With the increasing attention to teacher emotions, this study contributes to the research on teacher emotions in the context of educational reform. It provides actionable insights to help EFL teachers regulate their emotions and universities to establish effective support systems, contributing to the development of positive teacher emotions and, by extension, improving teacher competence within digital contexts.

Nevertheless, the study had several limitations. First, it examined a broad range of emotions experienced by EFL teachers during educational digital transformation. Future research could focus on specific emotions, to provide a deeper understanding of their causes and consequences. Second, since about half of the participants were recruited from universities in Guangxi, a province in Southwest China, the findings may lack full representativeness of EFL teachers nationwide. Future studies should adopt nationwide sampling strategies to enhance the generalizability of the results. Third, although the qualitative analysis systematically identified antecedent themes corresponding to the quantitative findings, the present study did not employ inferential statistical methods (e.g., regression or SEM) to test the factor-emotion associations. Future research could build upon our qualitatively derived antecedent themes and validate these relationships using regression or SEM in larger samples.

## Data Availability

The raw data supporting the conclusions of this article will be made available by the authors, without undue reservation.

## Footnotes

1 Although Parrott's taxonomy identifies six primary emotions including surprise, we excluded this emotion in the subsequent teacher emotion scale design and analysis. The reasons are as follows: First, the secondary emotion under the primary emotion of surprise is still surprise, so this category lacks sufficient explanatory richness.Second, surprise cannot be clearly categorized as either positive or negative, making valence analysis infeasible in our discussion.Third, prior scholars who adopted Parrott's framework (Chen, 2016; Han et al., 2023) excluded surprise when constructing their teacher emotion scales.
